# Parkinson disease-linked *GBA* mutation effects reversed by molecular chaperones in human cell and fly models

**DOI:** 10.1038/srep31380

**Published:** 2016-08-19

**Authors:** Alvaro Sanchez-Martinez, Michelle Beavan, Matthew E. Gegg, Kai-Yin Chau, Alexander J. Whitworth, Anthony H. V. Schapira

**Affiliations:** 1 Department of Biomedical Sciences, University of Sheffield, Sheffield, S10 2TN, UK; 2 Medical Research Council Mitochondrial Biology Unit, Cambridge Biomedical Campus, Hills Road, Cambridge CB2 0XY, UK; 3 Department of Clinical Neuroscience, Institute of Neurology, University College London, London NW3 2PF, UK

## Abstract

*GBA* gene mutations are the greatest cause of Parkinson disease (PD). *GBA* encodes the lysosomal enzyme glucocerebrosidase (GCase) but the mechanisms by which loss of GCase contributes to PD remain unclear. Inhibition of autophagy and the generation of endoplasmic reticulum (ER) stress are both implicated. Mutant GCase can unfold in the ER and be degraded via the unfolded protein response, activating ER stress and reducing lysosomal GCase. Small molecule chaperones that cross the blood brain barrier help mutant GCase refold and traffic correctly to lysosomes are putative treatments for PD. We treated fibroblast cells from PD patients with heterozygous *GBA* mutations and *Drosophila* expressing human wild-type, N370S and L444P *GBA* with the molecular chaperones ambroxol and isofagomine. Both chaperones increased GCase levels and activity, but also *GBA* mRNA, in control and mutant *GBA* fibroblasts. Expression of mutated *GBA* in *Drosophila* resulted in dopaminergic neuronal loss, a progressive locomotor defect, abnormal aggregates in the ER and increased levels of the ER stress reporter Xbp1-EGFP. Treatment with both chaperones lowered ER stress and prevented the loss of motor function, providing proof of principle that small molecule chaperones can reverse mutant *GBA*-mediated ER stress *in vivo* and might prove effective for treating PD.

The *GBA* gene encodes the lysosomal enzyme glucocerebrosidase (GCase), which cleaves the sphingolipid glucosylceramide into glucose and ceramide. Homozygous mutations in the *GBA* gene cause Gaucher disease (GD), a lysosomal storage disorder^1^. The pathogenic features of GD are associated with the accumulation of glucosylceramide in lysosomes in several cell types, including macrophages and neurons.

Although occasional reports of GD with PD appeared some years ago^2^,^3^, the link between *GBA* mutations and PD was clearly established in 2009^4^. Both homozygous and heterozygous *GBA* mutations are associated with an approximately equal risk for the development of PD. PD patients with *GBA* mutations tend to have an earlier age of onset and greater cognitive decline^4^,^5^,^6^,^7^. GCase activity is also significantly decreased in the substantia nigra and anterior cingulate cortex of sporadic PD brains^8^,^9^,^10^.

Lewy bodies are α-synuclein rich neuronal protein aggregates and are a pathological hallmark of PD. Impairment of the autophagy-lysosomal pathway (ALP) is implicated in the abnormal accumulation of α-synuclein^11^,^12^,^13^. In cellular and animal models where GCase is knocked down, knocked out or which express pathogenic mutations, α-synuclein is found to accumulate, exhibit properties of Lewy bodies (proteinase K resistant; ubiquitin positive) and be co-incident with impairment of the ALP^14^,^15^,^16^,^17^. ALP inhibition has also been implicated with mitochondrial dysfunction observed in *Gba* −/− and *gba* −/− mice and zebrafish^17^,^18^. Zebrafish lacking *gba* also exhibit loss of dopaminergic neurons, which occurs in the absence of α-synuclein^18^.

However, the exact mechanism by which GCase deficiency contributes to PD pathogenesis is unclear but may include the accumulation of α-synuclein, impaired lysosomal function and endoplasmic reticulum (ER) associated stress^19^. Accumulation of glucosylceramide in lysosomes may contribute to lysosomal dysfunction for homozygous *GBA* mutations but no evidence of glucosylceramide accumulation in PD brains with heterozygous *GBA* mutations has been reported^20^.

The two most common *GBA* mutations associated with PD are N370S and L444P^21^. These mutations have been reported to unfold in the ER^22^,^23^ and activate the unfolded protein response (UPR). There are three arms of the UPR: IRE1, PERK and ATF6. These proteins down-regulate protein translation, while enhancing the expression of ER chaperones, with the aim of decreasing the protein burden in the ER and refolding the proteins that have activated the UPR^24^. GCase that cannot be refolded by chaperones is retro-translocated to the cytoplasm and degraded by the ubiquitin-proteasome system^25^. Persistent activation of the UPR results in ER stress, with dysregulation of calcium and activation of apoptosis, and is implicated in several neurodegenerative disorders including PD^8^,^12^,^24^.

Therefore, *GBA* mutations in addition to impairing ALP in PD, may also elicit a gain of function by activating ER stress because the mutant protein is trapped in the ER. Markers of ER stress are elevated in PD brains with *GBA* mutations^8^ and dysregulation of ER calcium stores have been reported in cell models containing *GBA* mutations associated with PD^16^,^26^.

Enzyme replacement therapy is an effective treatment for type I GD, but cannot cross the blood brain barrier. Importantly, viral expression of wild-type *GBA* in the brains of GD mouse models has been shown to reduce α-synuclein pathology, restore memory deficits and protect dopaminergic neurons^15^,^27^,^28^. However, this requires injection into the brain and does not combat the GCase trapped in the ER. A more attractive approach is the use of small molecule chaperones that can cross the blood brain barrier, bind to GCase, and promote proper folding and delivery to lysosomes. Two chaperones that have been found to bind GCase and improve trafficking to the lysosome in GD fibroblasts are ambroxol and isofagomine^29^,^30^,^31^. Previously we have reported that ambroxol can increase GCase activity in GD fibroblasts and fibroblasts with heterozygous *GBA* mutations from subjects with and without PD^32^. We have expanded our data on fibroblasts from *GBA* mutation carriers and investigated whether isofagomine has similar effects to ambroxol.

*Drosophila melanogaster* models of PD have proven useful for dissecting the pathogenesis of disease^33^, although *Drosophila* do not have a homolog of α-synuclein. We have generated fly lines expressing human wild type or mutant N370S and L444P *GBA* to assess the contribution of ER stress to disease pathogenesis in the absence of the well documented GCase–α-synuclein bidirectional relationship^8^,^14^,^16^,^27^. These *Drosophila* lines were also used to assess the efficacy of ambroxol and isofagomine on ER stress *in vivo*.

Similar to other reports, we found that expression of *GBA* variants induces ER stress and progressive locomotor deficits^34^,^35^, as well as a modest loss of dopaminergic neurons. Feeding flies ambroxol or isofagomine alleviated ER stress and prevented the locomotor deficits. Hence, this model system provides evidence that *GBA* mutations cause aberrant ER stress which contributes to the pathogenic process but which can be alleviated by administration of molecular chaperones.

## Results

### Ambroxol and isofagomine increase GCase mRNA and enzyme activity in fibroblasts

**Figure 1:**
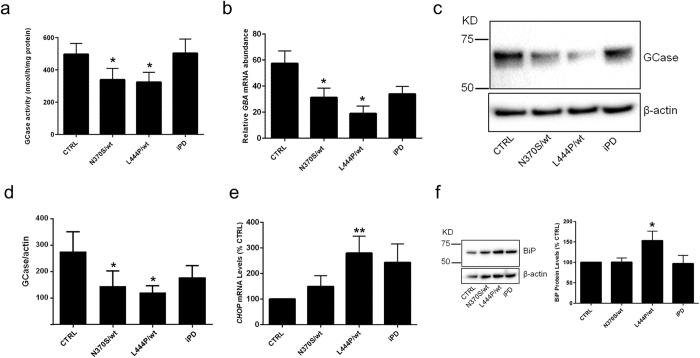
Expression and activity of GCase decreased in fibroblasts from Parkinson patients with heterozygous *GBA* mutations. (**a**) Analysis of GCase activity (n = 5 different cell lines/group) in fibroblasts from control subjects (wt/wt; CTRL), Parkinson subjects with N370S/wt, L444P/wt *GBA* mutations or idiopathic PD (wt/wt; iPD). (**b**) qRT-PCR analysis of *GBA* mRNA levels in indicated fibroblasts (n = 3 cell lines/group). (**c**) Immunoblot of fibroblasts for GCase protein expression. Protein loading normalized against β-actin. (**d**) Densitometric analysis of immunoblots (n = 5 cell lines/group). (**e**) qRT-PCR analysis of *CHOP* mRNA in fibroblasts (n = 7). (**f**) Immunoblot analysis of BiP protein levels in fibroblasts (n = 6). Protein loading normalised against β-actin. Data expressed as % CTRL fibroblasts. Quantitative data indicate means ± S.E.M. from indicated biological replicates. One-way ANOVA with Bonferroni’s correction was applied across the different groups (*P < *0*.05, **P < *0*.*01* versus CTRL).

Human dermal fibroblasts express barely detectable levels of α-synuclein compared to neurons (Supplementary Fig. 1). We therefore chose fibroblasts to investigate small molecule chaperones on mutant *GBA*, to be confident that any effects observed on ER stress were not due to the contribution of the well characterised relationship between perturbed α-synuclein homeostasis and mutant GCase reported in neurons and brain^14^,^15^,^16^. GCase activity was measured in control fibroblasts (wt/wt; CTRL), fibroblasts from PD patients with heterozygous *GBA* mutations (N370S/wt and L444P/wt) and fibroblasts from idiopathic (sporadic) PD patients (iPD). GCase activity was significantly decreased in N370S/wt and L444P/wt fibroblasts by 32% and 35%, respectively (P < 0.05 vs. CTRL; Fig. 1a). No loss of GCase activity was found in iPD fibroblasts. Similar to previous reports^32^,^36^ this decrease in GCase activity was concomitant with a significant decrease in both *GBA* mRNA and GCase protein levels in mutant *GBA* lines (P < 0.05 vs. CTRL; Fig. 1b–d). Under basal conditions, markers of UPR stress such as increased levels of *CHOP* mRNA (transcriptionally induced by both PERK and ATF6 arms of the UPR^24^) and protein levels of the chaperone BiP were significantly increased in L444P/wt by 280% (P < 0.01 vs. CTRL) and 153% (P < 0.05 vs. CTRL), respectively (Fig. 1e,f). No markers of the UPR were significantly increased in the N370S/wt or iPD lines.

**Figure 2:**
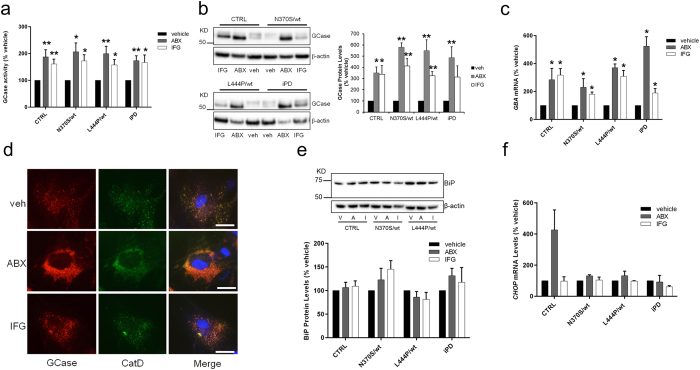
Ambroxol and isofagomine treatment increase GCase activity, protein levels and *GBA* mRNA in fibroblasts. (**a**) GCase activity in fibroblasts following 6 day treatment with vehicle (veh, black), 60 μM ambroxol (ABX, grey) or 50 μM isofagomine (IFG, white). Data are expressed as % GCase activity compared to respective vehicle treated cell line (n = 3). (**b**) Immunoblot for GCase protein expression following treatment with vehicle, ABX or IFG. Protein loading normalized against β-actin. (**c**) qRT-PCR analysis of *GBA* mRNA levels in fibroblasts treated with veh, ABX or IFG. Data normalized against *β*-*actin* mRNA and data expressed as % of vehicle treatment for respective cell line (n = 3). (**d**) Immunofluorescence for GCase (red) and the lysosomal marker cathepsin D (CatD, green) in N370S/wt fibroblasts treated with vehicle, ABX or IFG. Nuclei were counterstained with DAPI (blue). Scale bar 3 μm. (**e**) Immunoblot for BiP protein levels in fibroblasts treated with vehicle (V), ambroxol (A) or isofagomine (I). Protein loading normalized against β-actin and data expressed as % of vehicle treatment for respective cell line (n = 4). (**f**) qRT-PCR analysis of *CHOP* mRNA levels in fibroblasts treated with veh, ABX or IFG. Data normalized against β-actin mRNA levels and data expressed as % of vehicle treatment for respective cell line (n = 3). Quantitative data indicate means ± S.E.M. from indicated biological replicates. One-way ANOVA with Bonferroni’s correction was applied across the different groups (*P < *0*.05, **P < *0*.*01* versus vehicle treatment of respective cell line).

Treatment of control, heterozygous *GBA* and iPD fibroblasts with the protein chaperones ambroxol (60 μM) or isofagomine (50 μM) increased GCase activity after 6 days of treatment (Fig. 2a). These doses have previously been reported to be effective at increasing GCase activity over this time^29^,^32^. GCase activity was significantly increased in CTRL, N370S/wt, L444P/wt and iPD treated with ambroxol by 188% (P < 0.01), 206% (P < 0.05), 199% (P < 0.01) and 174% (P < 0.01), respectively, when compared to vehicle treated cells (DMSO). Isofagomine increased GCase activity in CTRL, N370S/wt, L444P/wt and iPD by 161% (P < 0.01), 172% (P < 0.05), 158% (P < 0.05) and 166% (P < 0.05), respectively. Similarly, GCase protein expression and *GBA* mRNA levels were increased in all cell lines treated with ambroxol or isofagomine (Fig. 2b,c). Immunofluorescence of N370S/wt cells for GCase and the lysosomal enzyme Cathepsin D suggest that both ambroxol and isofagomine increase lysosomal GCase content (Fig. 2d). A similar immunofluorescence pattern was observed for CTRL, L444P/wt and iPD lines treated with ambroxol or isofagomine (data not shown).

BiP levels were decreased in L444P/wt cell lines when treated with ambroxol (86 ± 12% of vehicle treated cells) or isofagomine (81 ± 15% of vehicle treated cells), although not significantly (Fig. 2e). Neither chaperone treatment had any effect on *CHOP* levels in L444P/wt cells (Fig. 2f). Surprisingly, 60 μM ambroxol treatment considerably increased CHOP levels in CTRL cells. Titration of ambroxol indicated that 5 μM ambroxol treatments of control, N370S/wt and iPD cells was enough to significantly increase GCase activity (Supplementary Fig. 2), while not increasing *CHOP* levels in CTRL cells. However, this dose was not sufficient to increase GCase activity in L444P/wt cells.

### Generation of Drosophila expressing human WT, N370S or L44P GBA

**Figure 3:**
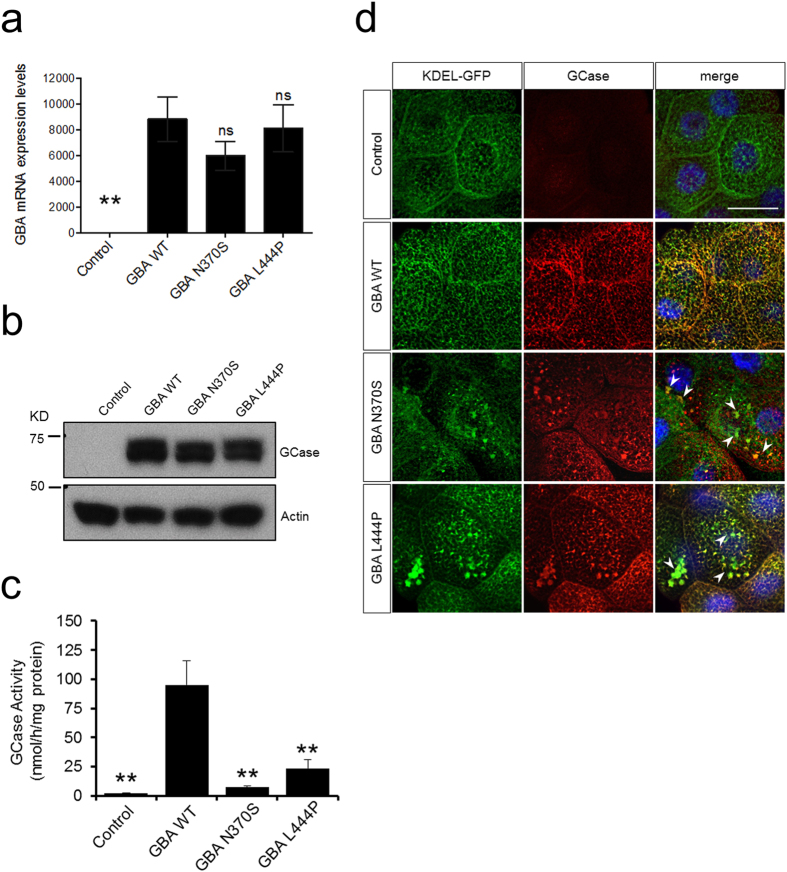
Expression of GBA variants co-localize with the endoplasmic reticulum. (**a**) qRT-PCR analysis of *GBA* mRNA levels in transgenic fly heads expressing *GBA* variants via *elav*-*GAL4*. (**b**) Immunoblot analysis of GBA protein levels in fly heads. (**c**) Analysis of GCase activity in heads of the indicated genotypes. (**d**) Immunostaining of anti-GCase (red) in salivary glands co-localizes with the ER marker KDEL-GFP (native GFP; green). DNA is marked with Hoechst (blue). Arrowheads indicated abnormal ER swelling. Scale bar 50 μm. Control genotypes are GAL4/+. Quantitative data indicate means ± S.E.M. from 3–4 biological replicates. One-way ANOVA with Bonferroni’s correction was applied across the different genotypes (**P < *0*.*01* vs. *GBA* wt; ns, non-significant).

To investigate further the impact of *GBA* mutations on ER stress and the potential protective effects of ambroxol and isofagomine *in vivo* we generated transgenic *Drosophila* to express human wt, N370S or L444P GCase. *GBA* mRNA levels and protein expression of human GCase were similar in the wt and mutant lines (Fig. 3a,b). Despite equivalent expression levels, the enzyme activity of GCase was significantly decreased (P < 0.01) by 82% in N370S flies and 75% in L444P flies compared to the wt line (Fig. 3c). Immunofluorescence revealed an abundant co-localisation of GCase with ER, however, unlike the wt, N370S and L444P caused abnormal aggregates and swellings within the ER (Fig. 3d).

**Figure 4:**
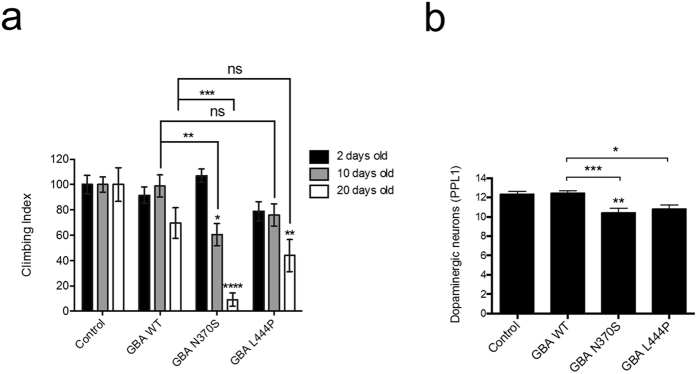
Analysis of neurodegeneration phenotypes upon GBA expression. Expression of GBA variants was induced by *elav*-*GAL4*, and flies were analyzed for (**a**) climbing ability and (**b**) dopaminergic neuron immunostaining with ageing. Statistical analysis of climbing assays used Kruskal-Wallis with Dunn’s multiple comparisons test, whereas one-way ANOVA with Bonferroni’s correction was used to analyze neuron loss (*P < *0*.05, **P < *0*.01, ***P < *0*.001, ****P < *0*.*0001*; ns, non-significant). Data represent mean ± SEM.

Pan-neuronal expression of the GCase variants did not significantly perturb life-span (Supplementary Fig. 3) or cause overt degeneration of the eye even after extensive aging (Supplementary Fig. 4). However, while expression of wt *GBA* had no effect on motor (climbing) ability, the two mutant *GBA* lines showed a progressive climbing defect (Fig. 4a). A significant decrease in climbing was detected after 10 days in N370S flies which deteriorated further by 20 days. The L444P line exhibited a significant decrease in climbing after 20 days. Furthermore, the number of dopaminergic neurons in both N370S and L444P flies after 30 days was significantly decreased compared to wt *GBA* flies (Fig. 4b).

**Figure 5:**
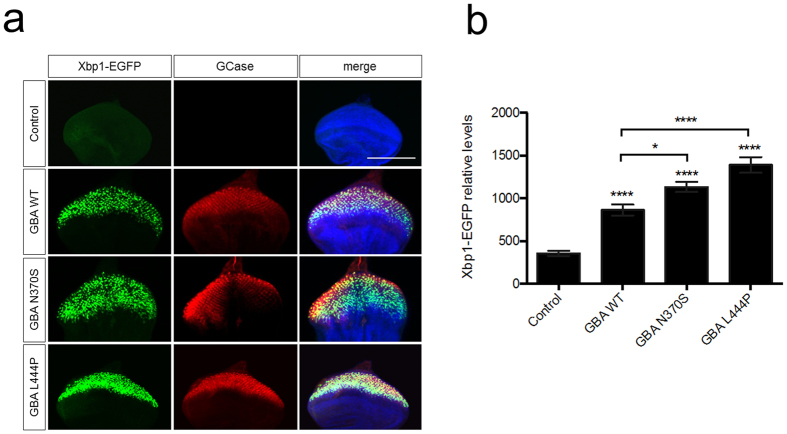
Expression of GBA variants increase ER stress. (**a**) Analysis of ER stress induction in eye imaginal discs expressing GBA variants via *GMR*-*GAL4*. Induction of ER stress was monitored by the ER stress reporter Xbp1-EGFP (green). GBA expression is indicated by anti-GBA immunostaining (red). DNA is marked with Hoechst (blue). (**b**) Quantification of Xbp1-EGFP levels compare to control (*GMR*-*GAL4/Xbp1*-*EGFP*). Scale bar 100 μm. Data represents means values from at least 10 imaginal discs. Statistical analysis was calculated using one-way ANOVA with Bonferroni’s correction (*P < *0*.05, ****P < *0*.*0001*). Data represent mean ± SEM.

To investigate the possible induction of ER stress we used a ER stress reporter transgene, Xbp1-EGFP, where GFP is only expressed following an ER stress-induced splice event^37^. Using the developing eye tissue as a tractable system, as previously reported^34^ expression of wt *GBA* resulted in increased Xbp1-EGFP levels compared to control (Fig. 5). However, expression of both N370S and L444P *GBA* induced significantly higher Xbp1-EGFP levels, relative to wt *GBA* flies (Fig. 5), indicating these forms caused an increased level of ER stress.

### Ambroxol and isofagomine reduce ER stress and reverse locomotor deficits in *Drosophila* with mutant GBA

**Figure 6:**
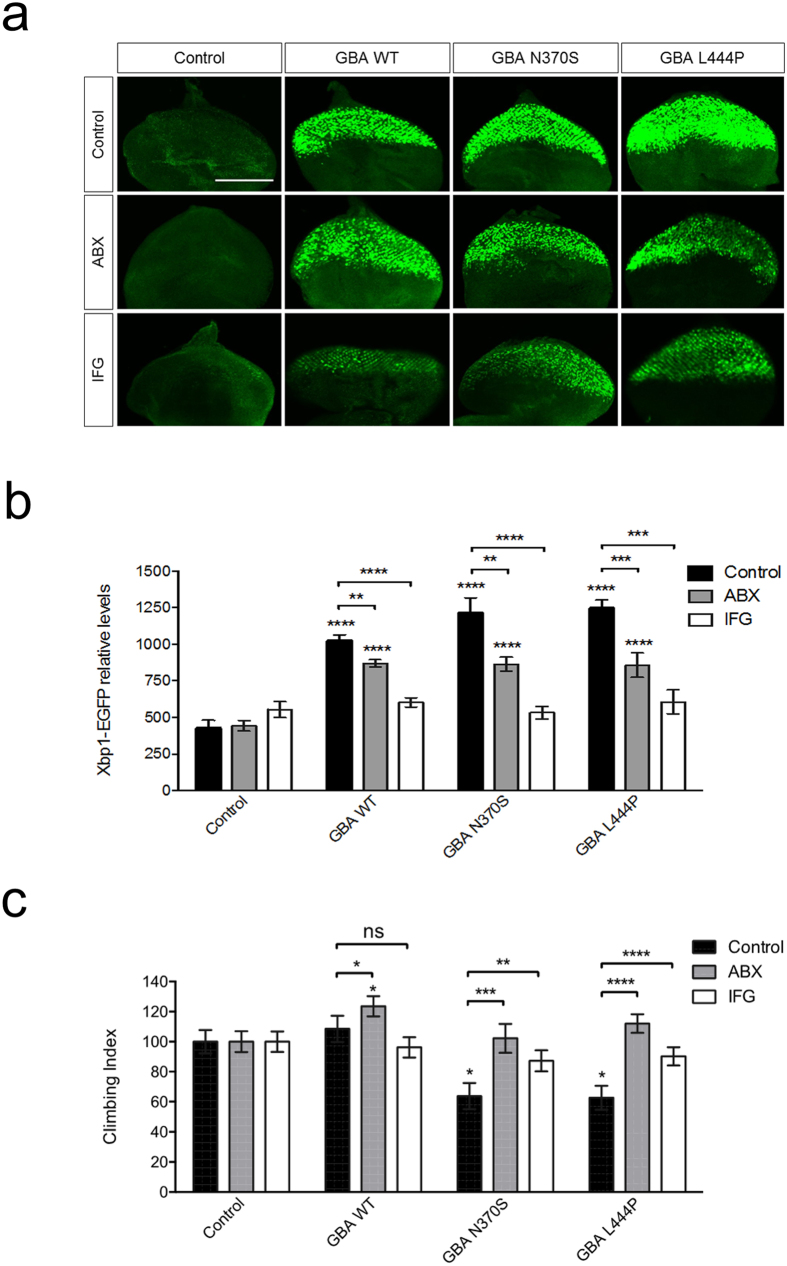
Ambroxol and isofagomine treatment reduce ER stress and rescue climbing deficits. (**a**) Analysis of ER stress induction in eye imaginal discs expressing GBA variants via *GMR*-*GAL4* in the presence or absence of ambroxol (500 μM) or isofagomine (50 μM) for 4 days. Induction of ER stress was monitored by the ER stress reporter Xbp1-EGFP (green). Scale bar 100 μm. (**b**) Quantification of Xbp1-EGFP levels in the indicated conditions. Data represents mean ± SEM from at least 10 imaginal discs. Statistical analysis was calculated using one-way ANOVA with Bonferroni’s correction. (**c**) Analysis of adult climbing ability in flies expressing GBA variants via *elav*-*GAL4* in the presence or absence of ambroxol (500 μM) or isofagomine (50 μM) for 10 days. Kruskal-Wallis with Dunn’s multiple comparisons test was used for analysis (*P < *0*.05, **P < *0*.01, ***P < *0*.001, ****P < *0*.*0001*; ns, non-significant). Data represent mean ± SEM.

*GBA* expressing flies were next raised on food containing ambroxol (500 μM) or isofagomine (50 μM). Isofagomine has been reported to bind more potently to GCase than ambroxol^29^,^38^,^39^. Therefore to reduce the chance of toxic side effects we used a lower dose of isofagomine. Exposure to either chaperone during development significantly reduced the levels of Xbp1-EGFP compared to untreated flies (Fig. 6a,b). Notably, treatment with isofagomine was able to reduce the high Xbp1-EGFP levels induced by all *GBA* variants back to levels comparable to control flies. Thus, these treatments were able to reverse the elevated ER stress *in vivo*.

To determine whether the reduction of ER stress by these chaperones provided a functional benefit to the flies, we assessed the locomotor ability of *GBA* expressing flies fed with ambroxol or isofagomine for 10 days. Strikingly, both chaperones were able to completely prevent the age-dependent decline in climbing ability caused by the expression of mutant *GBA* (Fig. 6c).

**Figure 7:**
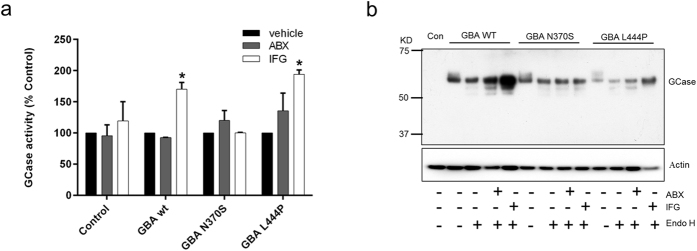
GCase activity and protein levels in wt, N370S and L444P flies following chaperone treatment. (**a**) GCase activity measured in flies treated with vehicle (veh, black), ambroxol (ABX, grey) or isofagomine (IFG, white) for 10 days. Data are expressed as % GCase activity compared to respective veh treated fly line. (**b**) Representative immunoblot for human GCase in respective fly lines in the absence or presence of ABX and IFG for 10 days. Lysates were treated with or without endoglycosidase H (endoH) prior to immunoblot. Data represent mean ± SEM (*P < 0.05 vs. respective vehicle treatment).

Isofagomine but not ambroxol significantly increased GCase activity by 170 ± 11% in wt flies (P < 0.05 versus vehicle treated wt flies) and 194 ± 7% in L444P flies (P < 0.05 versus vehicle treated L444P flies; Fig. 7a). The increased GCase activity following isofagomine treatment in wt and L444P flies was concomitant with an increase in protein levels (Fig. 7b). Treatment of human cell lysates with endoglycosidase H has been used to identify GCase species trapped in the ER^22^,^23^. Treatment of fly lysates with endoglycosidase H yielded the same pattern in wt and mutant *GBA* lines and was unaffected by chaperone treatment (Fig. 7b).

## Discussion

In this study we have used human cell lines and *Drosophila* models to investigate the biochemical consequences of the N370S and L444P *GBA* mutations that cause GD and significantly increase the risk for PD, and to evaluate the effects of two chaperones to reverse the abnormalities induced by these mutations. Our results confirm that the mutations cause reductions in GCase activity and elevated ER stress in the L444P fibroblasts and both the N370S and L444P flies. The flies exhibited reduced climbing activity and a loss of tyrosine hydroxylase positive neurons. Effects were partially reversed in flies by ambroxol and isofagomine.

Similar to other studies, we find that treatment of human dermal fibroblasts for several days with ambroxol or isofagomine increases GCase activity, protein expression and co-localisation with lysosomes^29^,^30^,^32^,^36^,^40^. Furthermore, this increase in GCase activity and protein expression occurs in control fibroblasts and fibroblasts from idiopathic PD to a similar extent as fibroblasts from PD patients with heterozygous *GBA* mutations or GD fibroblasts^32^,^36^. Both ambroxol and isofagomine treatment of fibroblasts increased *GBA* mRNA levels suggesting that these compounds elicit cellular responses to promote GCase expression in addition to their chaperoning ability. We opted to investigate treatment of cells with ambroxol or isofagomine over several days as we anticipate that this is the period of time required to re-fold GCase and for increased GCase trafficking to lysosomes to elicit measurable improvements in lysosomal function. Macrophages derived from inducible pluripotent stem cells (iPSC) from GD patients required five days of ambroxol or isofagomine treatment to reduce production of proinflammatory cytokines and improve the clearance of phagocytosed red blood cells^41^. Furthermore, ALP inhibition and perturbed α-synuclein homeostasis observed in dopaminergic neurons derived from iPSC from PD patients with *GBA* mutations^16^,^42^ require several days of small molecule chaperone treatment to be reversed (Yang *et al*., manuscript submitted).

Ambroxol treatment has been shown in fibroblasts to induce the coordinated lysosomal expression and regulation (CLEAR) network mediated via transcription factor EB^32^. Activation of the CLEAR network increases lysosomal biogenesis, clearance of damaged proteins and organelles by macroautophagy and maintains mitochondrial homeostasis^43^,^44^,^45^,^46^. In addition to increased GCase activity and expression levels, ambroxol treatment has been reported to increase the expression/activity of lysosomal cathepsins, the GCase transporter LIMP2, and saposin C, the endogenous activator of lysosomal GCase activity^32^,^36^. Recently, ambroxol has been reported to affect calcium homeostasis in lung type II pneumocytes^47^, which if applicable to other cell types, would modulate many cellular pathways.

Further studies on different ambroxol concentrations and duration of treatment are needed to dissect the physiological actions of this chaperone. The beneficial effects of ambroxol on GCase activity, lysosomal localisation, and inflammatory mediators have typically used concentrations of 50–100 μM^30^,^32^,^36^,^41^. The *K*_*m*_ and *K*_*i*_ of GCase for ambroxol at pH 7.0 has been reported to be about 5–10 μM^30^,^38^, while the current therapeutic doses of ambroxol (90–120 mg/day) yield a plasma drug concentration of 0.6–1.2 μM^47^. We found that 5 μM ambroxol significantly increased GCase activity in control, N370S/wt and iPD fibroblasts, and was not accompanied by increased *CHOP* mRNA levels seen in control fibroblasts at the higher dose. Unlike 100 μM ambroxol, 1 μM does not mobilise calcium^47^. However, higher doses may be required for the L444P GCase mutation as 5 μM was ineffective at increasing GCase activity, and has previously been shown to require higher doses (50 μM) to increase GCase activity by 30% and reduce ER trapping in L444P/L444P GD fibroblasts^31^. It is apparent from this and other studies that further characterization of the effects of ambroxol and isofagomine on ER stress makers in human models for the common *GBA* mutations is required.

Transgenic expression of human *GBA* in *Drosophila* generated substantial GCase activity, compared to background endogenous levels. However, the expression of N370S and L444P GCase resulted in significantly less GCase activity, increased ER stress, progressive loss of locomotor ability and the loss of dopaminergic neurons. Importantly, since *Drosophila* do not express endogenous α-synuclein, these findings show that mutant GCase mediated-ER stress is capable of causing loss of dopaminergic neurons in the absence of α-synuclein toxicity. Human and mouse models of pathogenic GCase have previously shown phenotypes relevant to PD, but because of the concomitant inhibition of the ALP and subsequent accumulation of α-synuclein, it was not possible to differentiate between the deleterious contribution of ER stress and autophagy impairment^14^,^15^,^16^,^42^. ER stress has been observed in other *Drosophila* models expressing human pathogenic mutant GCase, such as N370S, L444P and R120W, Rec*Ncil*^34^,^35^. During preparation of this manuscript ambroxol has also been shown to partially reverse the ER stress and loss of dopaminergic neurons in *Drosophila* expressing N370S and L444P GBA^48^. We have extended these observations by showing that isofagomine, as well as ambroxol^34^, can reverse the ER stress, and importantly, that both these chaperones can rescue locomotor deficits. Recently, deletion of the *GBA* homolog *dGBA1b* in *Drosophila* has been shown to affect locomotor ability and decrease lifespan^49^. Ubiquitin deposits and accumulation of Ref(2)P, the *Drosophila* homolog of p62/SQSTM1, also occurred, suggestive of impaired autophagy/quality control mechanisms. However, no change in dopaminergic neuronal number was observed, although evidence of other neurodegeneration was detected^49^.

The mechanism by which ambroxol and isofagomine exert these effects is not clear. We did observe a significant increase in GCase activity in the wt and L444P fly models following isofagomine treatment, while ambroxol had no significant effect on either wt or mutant GCase. It appears increasingly likely that these compounds may have multiple effects that may include the chaperoning of mutant GCase trapped in the ER, reversal of ER stress and transcriptional activation. The latter property may be most clearly reflected in our human-derived cell model where increases in *GBA* mRNA, protein levels and activity are seen even in the presence of only wt alleles. The N370S *GBA* mutant fly model response to treatment without a significant increase in GCase activity may in turn reflect effects on the reversal of ER stress and as yet unidentified additional mechanisms. In this respect it is notable that isofagomine was found to be protective in a neuronopathic GD mouse model by preventing the dysregulation of several mRNA and miRNA species in the brain involved in several pathways including neuroinflammation, mitochondrial function and axon guidance^50^. This protection occurred despite isofagomine not reducing the accumulation of substrate.

Regardless of the mechanism, reversal of ER stress and full restoration of locomotor function in our fly models by small molecule chaperones is an important proof of principal. Data suggest that the wild-type GCase becomes trapped in the ER and trafficking to lysosomes becomes impaired when cellular α-synuclein levels are elevated^8^,^9^,^14^. It is envisaged that if wild-type/mutant GCase can be refolded in the ER of humans, reducing ER stress and improving the trafficking of GCase to lysosomes, this will have important benefits not only for neuronopathic forms of GD (Type 2 and Type 3), for which no therapy is currently licensed, and PD patients with *GBA* mutations, but also sporadic forms of PD. Increased lysosomal GCase will help to reduce the accumulation of substrate in type 2 and type 3 GD, while in PD this should improve autophagy, thus reducing the formation of pathogenic α-synuclein species with a beneficial effect on disease pathogenesis^51^.

## Materials and Methods

### Fibroblast tissue culture lines

Human dermal fibroblasts were generated from skin biopsies taken from five controls (wt/wt), five PD patients with N370S/wt *GBA* mutations, five PD patients with L444P/wt *GBA* mutations and five patients with idiopathic PD. Controls were age-matched to PD patients and mutations were confirmed by Sanger sequencing. Fibroblasts were cultured in Dulbecco’s modified Eagle media (4.5 g/L glucose) supplemented with 10% (v/v) fetal calf serum, 1 mM sodium pyruvate and penicillin-streptomycin. Fibroblasts were not used after passage 12. All biopsies were obtained following informed patient consent following Hampstead Research Ethical committee approval (10/H0720/21/21). The methods were carried out in accordance with the relevant guidelines in the above-mentioned ethics approval.

### Chaperone treatment of fibroblasts

Treatment of fibroblasts with ambroxol (5–60 μM; Sigma-Aldrich A9797) or isofagomine (D-tartate (50 μM; Cayman Chemical 16137) started when cells were 50% confluent in 10 cm plates. Fibroblasts were treated with vehicle (dimethyl sulfoxide) or respective chaperone on days 0, 2, and 4. Fibroblasts were harvested on day 6 by trypsinisation and washed once in phosphate buffered saline (PBS) prior to freezing.

### Measurement of GCase activity

GCase activity was measured in fibroblast cell lysates (~20 μg protein) in McIlvaine citrate-phosphate buffer (pH 5.4) with 10 mM sodium taurochlorate and 5 mM 4–methylumbelliferyl–β–D–glucopyranoside as substrate at 37 °C as previously described^8^. The reaction was stopped with 0.25 M glycine (pH 10.4) and 4–methylumbelliferone fluorescence measured on a plate reader (excitation, 360 nm; emission 460 nm). Data were expressed as nmol 4–methylumbelliferone/h/mg protein.

*Drosophila* (~20) were homogenised in 200 μl RIPA buffer (150 mM NaCl, 1% (v/v) NP-40, 1% (w/v) sodium deoxycholate, 0.1% (w/v) SDS, 50 mM Tris, pH 8). Debris was pelleted and homogenate diluted in H_2_O to have a 2 mg/ml protein concentration. An aliquot (20 μg protein) was assayed as above. GCase activity was completely abolished in the presence of the GCase inhibitor conduritol B epoxide (10 mM). Note that GCase activity measured in RIPA buffer yields similar results as lysates prepared in 1% (v/v) TX-100 in PBS.

### Immunoblotting

Fibroblasts were lysed in 1% (v/v) Triton X-100 in PBS supplemented with protease inhibitors. For *Drosophila* samples, immunoblotting was done following standard protocols as described previously^52^. Proteins samples were isolated from whole adult fly heads. Protein lysates (20 μg) were resolved by SDS-PAGE and transferred to Hybond P (GE Healthcare). Blots were probed with antibodies against human GCase (clone 2E2, 1:1000, Merck Millipore), BiP (abcam, ab21685) or β-actin (human: abcam, ab82618; *Drosophila*: clone C4, 1:10000, Merck Millipore). Bands were detected with respective horse radish peroxidase-linked secondary antibodies (Dako) and Luminata Forte enhanced chemiluminescence (Merck Millipore). Density of bands was determined using Image Lab software 5.1 (Bio-Rad).

### Endoglycosidase H treatment of fly lysates

Fly lysates were prepared as for immunoblotting and 20 μg protein treated with 1000 units of endoglycosidase H (New England Biolabs) according to manufacturer’s instructions for 2 hours at 37 °C. Reaction was stopped by addition of gel loading dye and lysates immunoblotted for human GCase as above.

### Quantitative real time PCR

For cell samples, RNA was extracted from fibroblasts using RNeasy mini kit (Qiagen) and converted to cDNA (QuantiTect, Qiagen or nanoscript, Primer Design) and relative mRNA levels for *GBA* and *β*-*actin* were measured using TaqMan assays (*GBA*, Hs00986836_g1; *β*-*actin*, Hs99999903_m1; Applied Biosystems) using a STEP One PCR machine (Applied Biosystems). *CHOP* mRNA levels were measured using QuantiTect SYBRgreen (Qiagen) using the following primers: *CHOP*, ACC AAG GGA GAA CCA GGA AAC G, TCA CCA TTC GGT CAA TCA GAG C; *β*-*actin*, TCT ACA ATG AGC TGC GTG TG, GGT GAG GAT CTT CAT GAG GT. Relative *GBA* and *CHOP* mRNA levels were calculated using the 2^−ΔΔCT^ method and normalised against *β*-*actin*.

For *Drosophila* samples, RNA was extracted using a RNeasy RNA purification kit (Qiagen), and cDNA was synthesized using a Protoscript II first-strand cDNA synthesis kit (New England BioLabs) according to the manufacturers’ instructions. Relative GBA mRNA levels were calculated using the 2^−ΔΔCT^ method and normalized to the ribosomal reference gene, *RNA18S*. The primers used for *Drosophila* qRT-PCR were as follows: *GBA*: TGGGCAGTGACAGCTGAA, CTGGAAGGGGTATCCACTCA; *RNA18S*: TCTAGCAATATGAGATTGAGCAATAAG, AATACACGTTGATACTTTCATTGTAGC.

### Immunofluorescence

Fibroblasts were treated as above with chaperones. Cells were fixed with 3.7% paraformaldehyde (PFA), permeabilised with methanol and then 0.3% (v/v) Triton X-100 in phosphate buffered saline, and blocked in BlockAce (BioRad). Permeablised cells were incubated overnight (4 °C) with anti-GCase antibody (clone 2E2, 1:100, Merck Millipore) and anti-cathepsin D antibody (clone EPR3057Y, 1:100, abcam). Respective fluorescent secondary antibodies (alexa fluor-488/594, 1:100, Invitrogen) were used for detection and coverslips mounted in CitiFluor containing DAPI.

Salivary glands and eye imaginal discs were dissected from third instar larvae and fixed with 4% PFA/PBS solution for 20 min at room temperature, then permeabilised using 0.1% Triton X-100/PBS solution for 3 × 10 min. Samples were blocked for 1 h in 1% bovine serum albumin/PBS solution before addition of the primary antibody for 2 h. Antibodies used were: mouse anti-hGBA (clone 2E2, 1:100, Merck Millipore). AlexaFluor 488 and 568 secondary antibodies from Invitrogen (1:200) were added in 1% PBS for 2 h, then cell nuclei were stained with 0.1 μg/ml Hoechst for 5 min. *UAS*-*KDEL*-*EGFP* and *UAS*-*Xbp1*-*EGFP* were imaged using the native GFP. Fluorescence quantification was performed using ImageJ using the analyze particles function and measuring the area.

*Drosophila* brains were dissected from 30-day old flies and immunostained with anti-tyrosine hydroxylase (Immunostar Inc. 22491, 1:100) as described previously^53^. Brains were imaged with an Olympus FV1000 confocal with SIM-scanner on a BX61 upright microscope using a 63X oil 0.95 NA objective. Tyrosine hydroxylase-positive neurons were counted under blinded conditions.

### *Drosophila* stocks and procedures

*Drosophila* were raised under standard conditions at 25 °C (unless otherwise indicated) on agar, cornmeal and yeast food. *w*^*1118*^, *elav*-*GAL4, GMR*-*GAL4, fkh*-*GAL4, UAS*-*KDEL*-*GFP*, and *UAS*-*Xbp1*-*EGFP* strains were obtained from the Bloomington *Drosophila* Stock Center (Bloomington, IN). Transgenic lines expressing human *GBA (UAS*-*hGBA*) were constructed by cloning the entire open reading frame (WT, N370S and L444P) into pUAST-attB vector (BestGene Inc.). The ZH-51C attP site was used for phiC31 site-directed integration (Bloomington stock, 24482). For all integration events, multiple independent lines were initially isolated and assessed for consistent effects before selecting single lines for further analysis. Climbing assays were performed as previously described^54^. For all the experiments only males were used.

### Chaperone treatment of flies

For chaperone feeding, flies of the specified genotypes were maintained on standard media containing ambroxol hydrochloride (Sigma-Aldrich A9797) or isofagomine (D-tartrate) (Cayman Chemical 16137), dissolved in water, to final concentrations of 500 μM and 50 μM. For eye imaginal discs analysis, third instar larvae were raised in food containing drug (4 days feeding during larval stages), and imaginal discs were analyzed by immunohistochemistry and quantified using Image J. At least 10 discs were analyzed per genotype. For climbing assays at least 50 adult males collected from crosses raised on standard media were aged on food containing drug for 10 days before analyzing behavior.

### Life span

At least 100 recently emerged adult males were collected under light anesthesia and housed at a density of 25 males per vial. Flies were passaged every other day, and the number of dead flies was recorded.

### Light microscopy imaging

Light microscopy imaging was assessed using a Nikon motorized SMZ stereo zoom microscope fitted with 1x Apo lens. Extended focus images were then generated using Nikon Elements software, using the same settings for all the genotypes. Flies were anaesthetised during the process. All animals of a given genotype displayed essentially identical phenotypes and randomly selected representative images are shown.

### Statistical analysis

Calculations were performed using GraphPad Prism 6.0. Adult climbing analysis is not normally distributed so the data were analyzed using Kruskal-Wallis non-parametric test with Dunn’s correction for multiple comparisons. Protein and mRNA expression levels in fibroblasts and *Drosophila*, and dopaminergic neurons count significance was determined by one-way analysis of variance (ANOVA) with the Bonferroni’s multiple comparison test. For life span experiments Log-rank (Mantel-Cox) test was used for the analysis. Significance levels are indicated in figure legends. Unless specifically indicated, no significant difference was found between a sample and any of the other samples.

## Additional Information

**How to cite this article**: Sanchez-Martinez, A. *et al*. Parkinson disease-linked *GBA* mutation effects reversed by molecular chaperones in human cell and fly models. *Sci. Rep.* **6**, 31380; doi: 10.1038/srep31380 (2016).

## Supplementary Material

Supplementary Information
