# Supplementary material for: Parkinson disease-linked GBA mutation effects reversed by molecular chaperones in human cell and fly models
Source: Sci Rep. 2016 Aug 19;6:31380. doi: 10.1038/srep31380 (PMC4990939; doi:10.1038/srep31380)
Supplement: Supplementary Information [file srep31380-s1.pdf]

Parkinson disease-linked *GBA* mutation effects reversed by molecular chaperones in human cell and fly models

Alvaro Sanchez-Martinez[1,2 †], Michelle Beavan[3 †], Matthew E. Gegg[3 †], Kai-Yin Chau[3], Alexander J. Whitworth[1,2 *], Anthony H.V. Schapira[3 *]

1. Department of Biomedical Sciences, University of Sheffield, Sheffield, S10 2TN, UK.
2. Medical Research Council Mitochondrial Biology Unit, Cambridge Biomedical Campus, Hills Road, Cambridge CB2 0XY, UK
3. Department of Clinical Neuroscience, Institute of Neurology, University College London, London NW3 2PF, UK

[†]The authors wish it to be known that, in their opinion, the first three authors should be regarded as joint First Authors

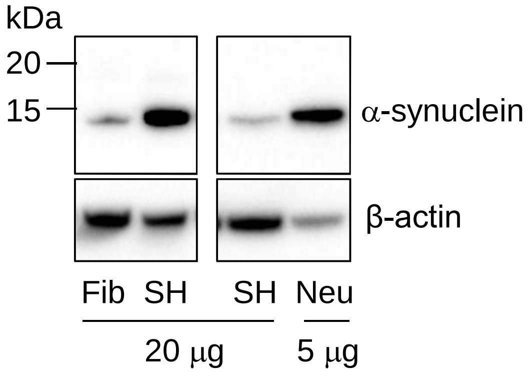

Supplementary Figure 1. Expression of α-synuclein in fibroblasts is very low. Human dermal fibroblasts (Fib), human neuroblastoma SH-SY5Y cell line (SH) and primary cortical mouse neurons (neu) were lysed in buffer containing 0.1% (w/v) SDS, 150 mM NaCl, 10 mM Tris (pH 7.5) and separated by SDS-PAGE. Proteins were transfered to Hybond P membrane (GE Healthcare), fixed for 30 minutes with 4% (v/v) paraformaldehyde, 0.01% (v/v) glutaraldehyde in PBS (pH 7.4)[1] and total α-synuclein levels measured by western blot using a mouse-monoclonal antibody that detects both human and mouse α-synuclein (abcam, clone 4D6). Human fibroblasts have barely detectable α-synuclein levels compared to SH-SY5Y cells, which in turn contain much less α-synuclein than primary mouse neurons. Note that only 5 μg of lysate was loaded for neurons compared to 20 μg for fibroblasts and SH-SY5Y cells. Fibroblasts had to be run on a separate blot to neurons to allow for the very weak signal in fibroblasts to be detected by ECL reagent.

[1] Sasaki A, Arawaka S, Sato H, Kato T. Sensitive western blotting for detection of endogenous Ser129-phosphorylated α-synuclein in intracellular and extracellular spaces. Sci Rep. 2015. 5:14211. doi: 10.1038/srep14211.

A

ABX (μM)	GCase Activity	GBA mRNA
vehicle	100	100
5	145	152
10	207	201
30	245	271

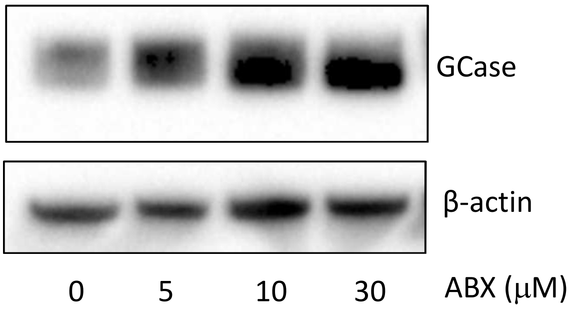

B

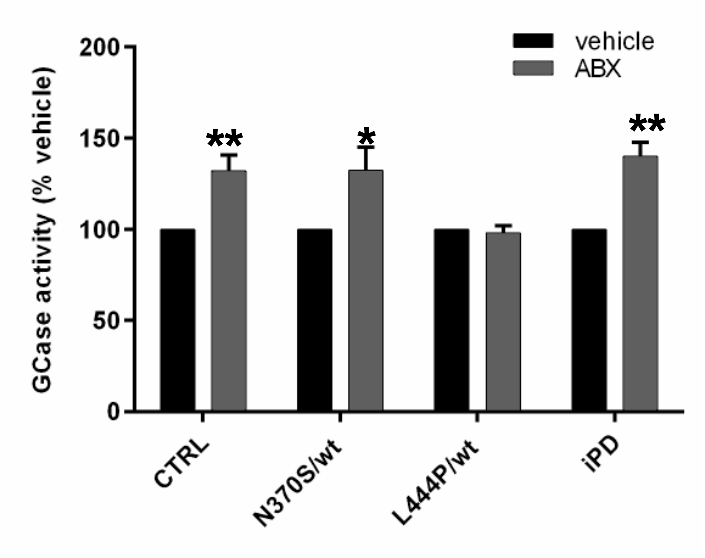

C

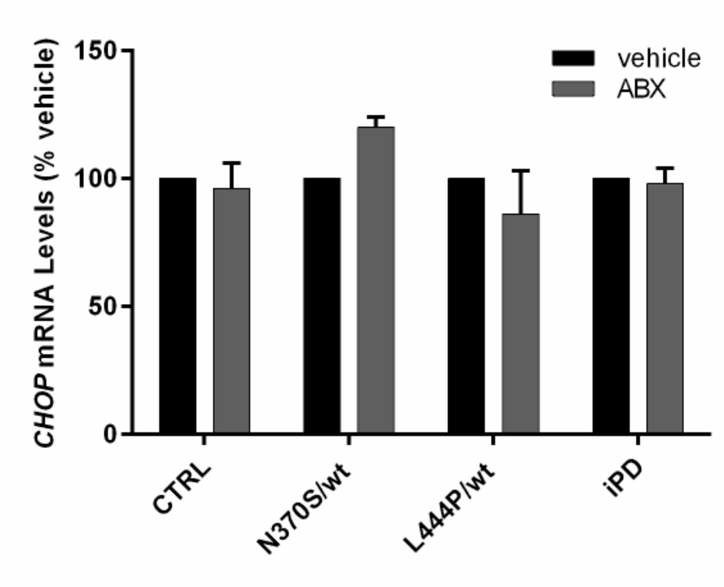

Supplementary Figure 2. The effect of lower ambroxol concentrations on GCase expression and *CHOP* mRNA. (A) Control fibroblasts (wt/wt) were treated with 0 – 30 μM ambroxol (ABX) for 4 days (treatment on days 0 and 2) and GCase activity and *GBA* mRNA levels measured. Data normalised against protein concentration and *β-actin* mRNA levels for activity and qRT-PCR analysis, respectively. Data expressed as % of vehicle treated. Representative immunoblot for GCase showing increased protein expression with ambroxol treatment. (B) Treatment of CTRL, N370S/wt, L444P/wt or iPD fibroblasts with vehicle (veh) or 5 μM ambroxol (ABX) for 4 days. GCase activity normalised against protein concentration and expressed as % of vehicle treated cells for each cell line. * $P < 0.05$ vs. respective vehicle control; ** $P < 0.05$ vs. respective vehicle control using one-tailed t-test. (C) qRT-PCR analysis of *CHOP* mRNA levels in fibroblasts treated with veh or 5 μM ABX. Data normalized against β-actin mRNA levels and data expressed as % of vehicle treatment for respective cell line (n=3).

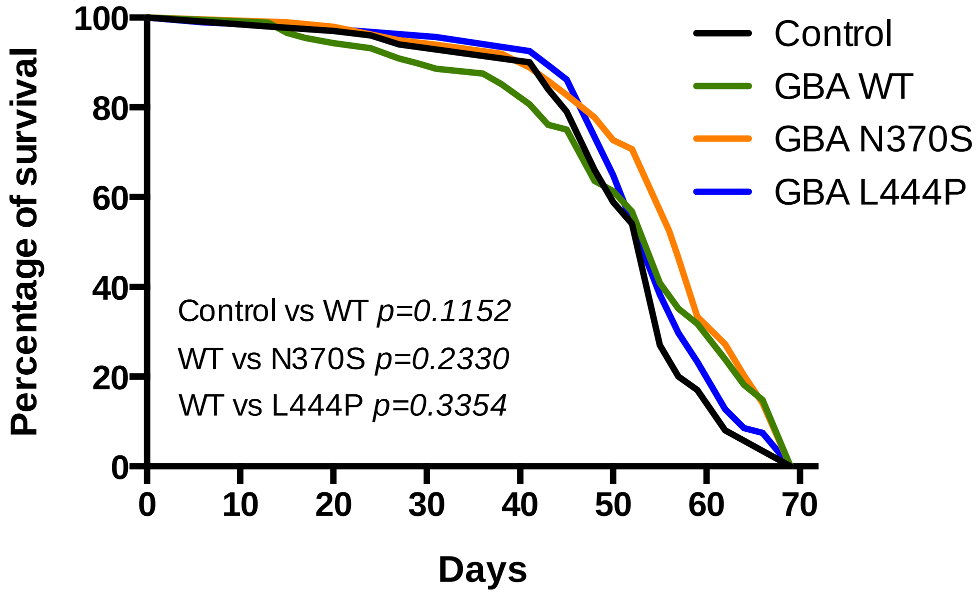

Supplementary Figure 3. Expression of *GBA* variants with a pan-neural driver *elav-GAL4* does not alter life span.

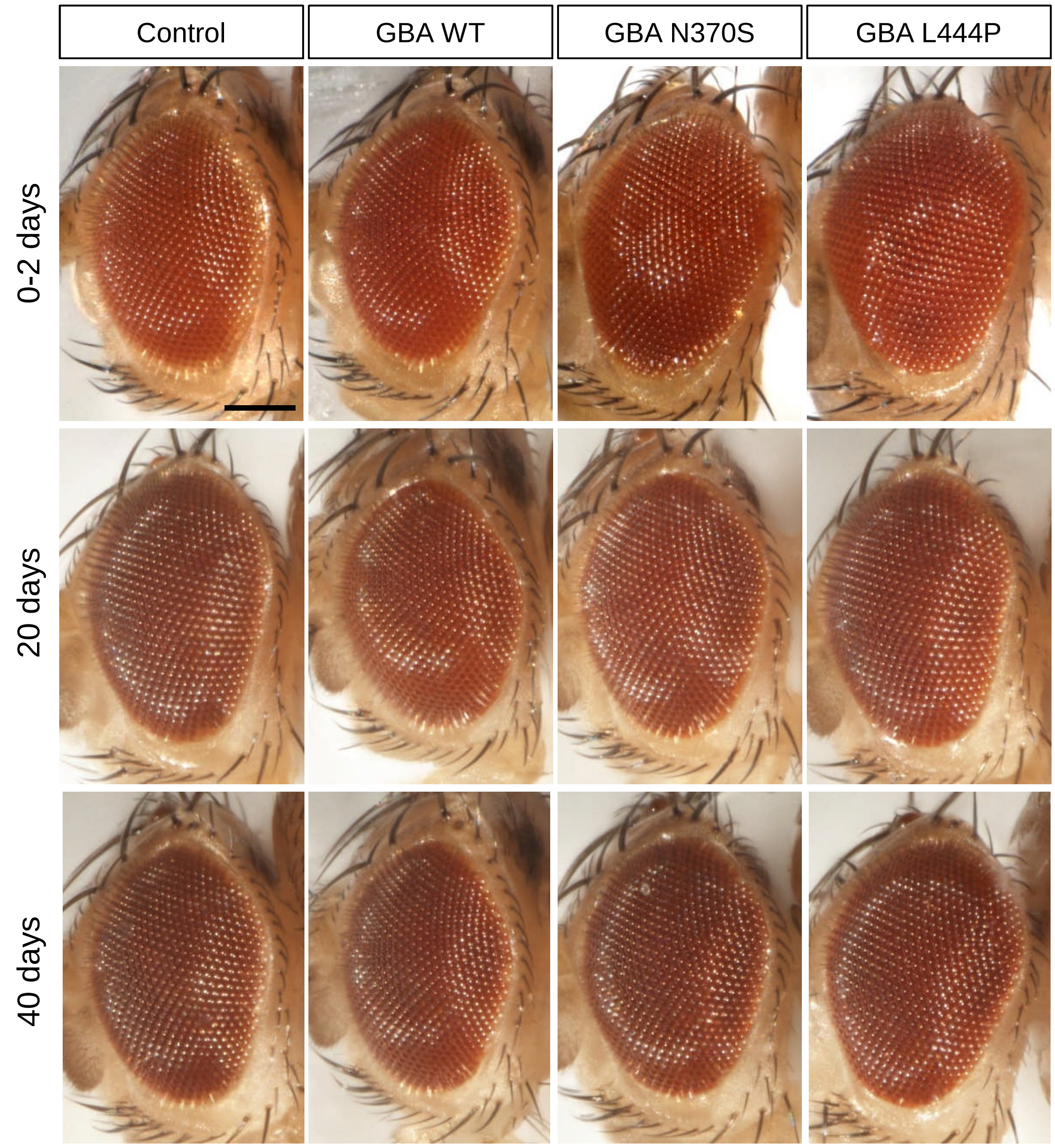

Supplementary Figure 4. Overexpression of *GBA* variants in adult eye using *GMR-GAL4* does not show an overt phenotype neither in young nor old flies.
